# Identification of an α(1→6) mannopyranosyltransferase (MptA), involved in *Corynebacterium glutamicum* lipomanann biosynthesis, and identification of its orthologue in *Mycobacterium tuberculosis*

**DOI:** 10.1111/j.1365-2958.2007.05884.x

**Published:** 2007-09

**Authors:** Arun K Mishra, Luke J Alderwick, Doris Rittmann, Raju V V Tatituri, Jerome Nigou, Martine Gilleron, Lothar Eggeling, Gurdyal S Besra

**Affiliations:** 1 School of Biosciences, University of Birmingham Edgbaston, Birmingham B15 2TT, UK; 2 Institute for Biotechnology 1, Research Centre Juelich D-52425 Juelich, Germany; 3 Institute de Parmacologie et de Biologie Structurale UMR CNRS 5089, Toulouse, France

## Abstract

*Corynebacterium glutamicum* and *Mycobacterium tuberculosis* share a similar cell wall architecture, and the availability of their genome sequences has enabled the utilization of *C. glutamicum* as a model for the identification and study of, otherwise essential, mycobacterial genes involved in lipomannan (LM) and lipoarabinomannan (LAM) biosynthesis. We selected the putative glycosyltransferase-Rv2174 from *M. tuberculosis* and deleted its orthologue NCgl2093 from *C. glutamicum*. This resulted in the formation of a novel truncated lipomannan (Cg-t-LM) and a complete ablation of LM/LAM biosynthesis. Purification and characterization of Cg-t-LM revealed an overall decrease in molecular mass, a reduction of α(1→6) and α(1→2) glycosidic linkages illustrating a reduced degree of branching compared with wild-type LM. The deletion mutant's biochemical phenotype was fully complemented by either NCgl2093 or Rv2174. Furthermore, the use of a synthetic neoglycolipid acceptor in an *in vitro* cell-free assay utilizing the sugar donor β-d-mannopyranosyl-1-monophosphoryl-decaprenol together with the neoglycolipid acceptor α-d-Man*p-*(1→6)-α-d-Man*p-O*-C_8_ as a substrate, confirmed NCgl2093 and Rv2174 as an α(1→6) mannopyranosyltransferase (MptA), involved in the latter stages of the biosynthesis of the α(1→6) mannan core of LM. Altogether, these studies have identified a new mannosyltransferase, MptA, and they shed further light on the biosynthesis of LM/LAM in *Corynebacterianeae*.

## Introduction

The human pathogen and aetiological agent of tuberculosis, *Mycobacterium tuberculosis*, belongs to the distinct and unusual group of the *Corynebacterianeae*, which includes other human pathogens, such as *Mycobacterium leprae* and *Corynebacterium diphtheriae*, the causal agents of leprosy and diphtheria respectively (Coyle and Lipsky, 1990; Bloom and Murray, 1992). Furthermore, non-pathogenic bacteria also belong to this taxon, such as *Corynebacterium glutamicum*, which is used in the industrial production of amino acids (Stackebrandt *et al*., 1997; Sahm *et al*., 2000).

The unique cell wall ultrastructure that is common among these bacilli is composed of a mycolyl-arabinogalactan–peptidoglycan (mAGP) complex (Daffe *et al*., 1990; McNeil *et al*., 1990; 1991; Besra *et al*., 1995; Brennan, 2003; Dover *et al*., 2004). Moreover, the packing and ordering of mycolic acids and additional lipids within the outer envelope results in a highly impermeable barrier characteristic of this genera (Minnikin *et al*., 2002). Other cell wall-associated lipids, such as phosphatidyl-*myo*-inositol (PI) mannosides (PIMs) and lipoglycans, termed lipomannan (LM) and lipoarabinomannan (LAM), are also found in the outer leaflet of the cell wall (Hill and Ballou, 1966; Brennan and Ballou, 1967; 1968; Brennan and Nikaido, 1995; Besra *et al*., 1997; Morita *et al*., 2004). In addition to their physiological function and potential as drug targets, these glycoconjugates also play a key role in the modulation of the host response during infection (Schlesinger *et al*., 1994; Chatterjee and Khoo, 1998; Maeda *et al*., 2003; Nigou *et al*., 2002).

**Fig. 1:**
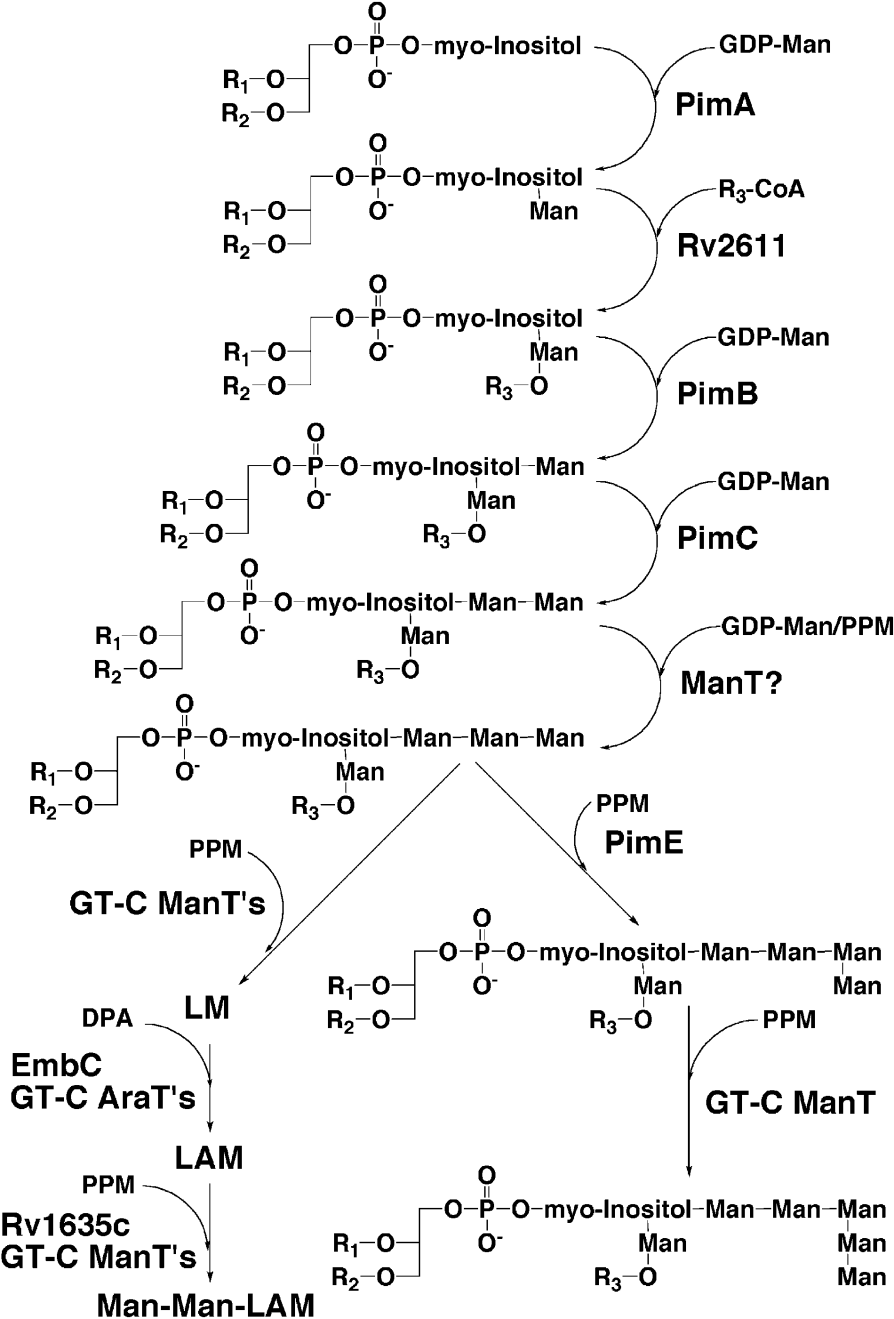
Proposed glycolipid biosynthetic pathway leading to LAM via LM and PIMs. ManT, mannosyltransferase; AraT, arabinosyltransferase; PPM, polyprenyl-1-monophosphoryl-mannose; DPA, decaprenyl-1-monophosphoryl-arabinose.

The current paradigm of mycobacterial lipoglycan biosynthesis follows a linear pathway, PI → PIM → LM → LAM (Besra and Brennan, 1997), with each individual step synthesizing an increasingly glycosylated molecule catalysed by discrete processive and non-processive glycosyltransferases (Fig. 1). PI acts as a substrate for the α-mannosyltransferase PimA (Rv2610c), which transfers a mannopyranosyl (Man*p*) residue from GDP-Mannose to the 2-position of PI to form PIM_1_ (Kordulakova *et al*., 2002). The second mannosylation step catalysed by PimB (Rv0557) may occur before, or after acylation of PIM_1_ by Rv2611c (Kordulakova *et al*., 2003), and results in the formation of Ac_1_PIM_2_ (Schaeffer *et al*., 1999). We have previously shown that PimB was also directly involved in synthesizing a novel mannosylated glycolipid, 1,2-di-*O*-C_16_/C_18:1_-(α-d-mannopyranosyl)-(1→4)-(α-d-glucopyranosyluronic acid)-(1→3)-glycerol (ManGlcAGroAc_2_) (Tatituri *et al*., 2007), which is indicative of a more complex biosynthetic pathway than previously considered. A third Man*p* residue is added by PimC to form Ac_1_PIM_3_ (Kremer *et al*., 2002). Recently, PimE (Rv1159) has been implicated in higher PIM biosynthesis and the synthesis of Ac_1_PIM_5_ (Morita *et al*., 2006); however, the enzyme responsible for the synthesis of the intermediate Ac_1_PIM_4_, from Ac_1_PIM_3_, remains elusive. Indeed, Ac_1_PIM_4_ is the likely precursor to LM formation (Morita *et al*., 2006). It has been proposed that at this point, a transition occurs from glycosyltransferases, utilizing nucleotide-derived sugar substrates characterized by the GT-A/B superfamily (Liu and Mushegian, 2003), to glycosyltransferases utilizing polyprenyl-phosphate sugars and the GT-C superfamily (Liu and Mushegian, 2003), for the elongation and branching of LM and LAM (Morita *et al*., 2006). More recently, Rv2181 has been reported to be involved in the synthesis of the α(1→2)-Man*p*-linked branches, characteristic of the mannan backbone in LM and LAM (Kaur *et al*., 2006). However, the enzyme required for the synthesis of the core linear LM/LAM mannan domain through an α(1→6) mannosyltransferase remains to be identified. The mature LM is then further glycosylated by the essential arabinofuranosyltransferase EmbC (G.S. Besra, unpubl. results), to form LAM (Berg *et al*., 2005), and recently a novel mannosyltransferase, MT1671, has been shown to add terminal Man*p* residues to the mature LAM in *M. tuberculosis* CDC1551 to form ManLAM (Dinadayala *et al*., 2006).

Comparative analyses of LAMs from various strains of mycobacteria have shown that, apart from the common core structure, various levels of ‘capping’ exists at the non-reducing terminus of the arabinan domain, in the form of AraLAM, ManLAM and PILAM (Chatterjee *et al*., 1993; Khoo *et al*., 1995; Guerardel *et al*., 2002). The presence or absence of terminal Man or insoitol (Ins) residues has emerged as the focal point for the current paradigm of immunomodulation (Nigou *et al*., 2002; 2003). ManLAMs have the capacity to inhibit the production of pro-inflammatory cytokines, such as IL-12 and TNF-α (Knutson *et al*., 1998; Nigou *et al*., 2002), whereas PILAM has the ability to inhibit the proliferation of these cytokines (Adams *et al*., 1993; Gilleron *et al*., 1997). Slow-growing mycobacteria, such as *M. tuberculosis* and *M. leprae*, exhibit a ManLAM phenotype and are able to exist and replicate within phagocytic cells. However, faster-growing strains such as *Mycobacterium smegmatis*, do not, which illustrates the importance of ManLAM as a key virulence factor (Nigou *et al*., 2002; 2003).

Apart from *C. glutamicum* and *M. tuberculosis* sharing a similar cell wall architecture, the availability of completed genome sequences for both organisms has enabled us to use *C. glutamicum* as a suitable model for the identification and functional study of mycobacterial genes involved in arabinogalactan and LAM biosynthesis (Gibson *et al*., 2003; Gande *et al*., 2004; Alderwick *et al*., 2005; 2006; Seidel *et al*., 2007a). In this article, we have examined one such open reading frame (ORF) from *C. glutamicum* NCgl2093, which encodes a putative GT-C glycosyltransferase on the basis of sequence similarity and homology alignment using *C. glutamicum* as a model to identify the key α(1→6) mannosyltransferase involved in LM biosynthesis and a potential new drug target.

## Results

### Genome comparison of the NCgl2093/Rv2174 locus

**Fig. 2:**
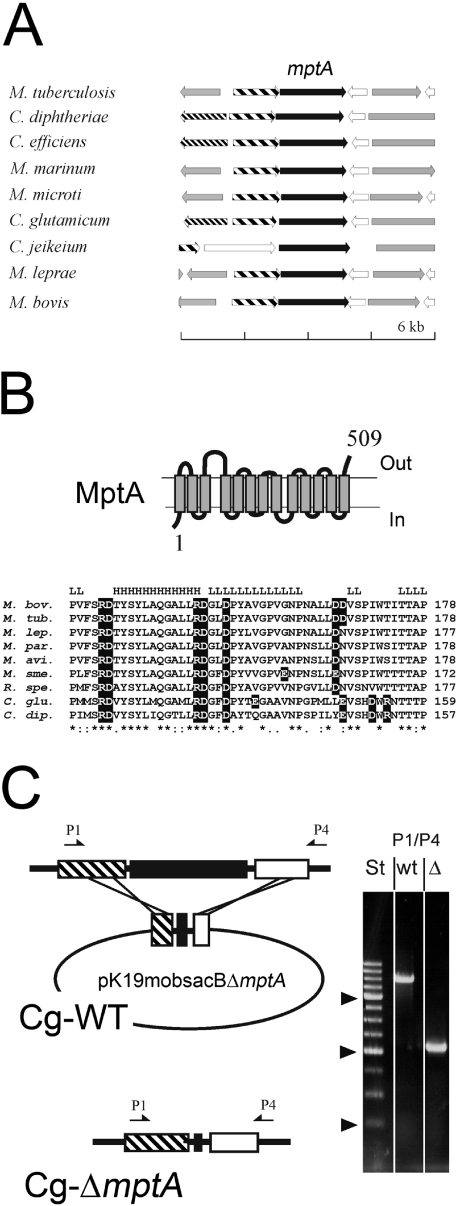
Comparison of the *mptA* locus within the *Corynebacterianeae* and in-frame deletion of Cg-mptA. A. The locus in the bacteria analysed consists of *mptA* which in *C. glutamicum* has the locus tag NCgl2093 and in *M. tuberculosis* Rv2174. Upstream of *mptA*, an ORF is conserved which could form together with *mptA* a transcriptional unit, and which is predicted to encode a polyprenyl synthetase. The genomic region displayed encompasses 6 kb, and orthologous genes are highlighted accordingly. B. MptA spans the membrane 13 times and a large loop connects TMH 3 and 4. Part of the loop sequence is given, where acid and basic residues are highlighted. On top of the sequence comparison the predicted secondary structure is given, with *H* indicating a helical structure, and *L* a loop region. The entire region has a high solvent accessibility, which indicates together with the conserved aspartyl residues their functional significance*. M. bov.*, *Mycobacterium bovis*; *M. tub*. *Mycobacterium tuberculosis*; *M. lep.*, *Mycobacterium leprae*; *M. par.*, *Mycobacterium paratuberculosis*; *M. avi*., *Mycobacterium avium*; *M. sme.*, *Mycobacterium smegmatis*; *R. spe.*, *Rhodococcus* sp. (strain *RHA1*); *C. glu.*, *Corynebacterium glutamicum*; *C. dip.*, *Corynebacterium diphtheriae*. C. Strategy to delete Cg-*mptA* using the deletion vector pK19mobsacBΔ*mptA*. This vector carries 18 nucleotides of the 5′ end of Cg-*mptA* and 36 nucleotides of its 3′ end thereby enabling the in-frame deletion of almost the entire Cg-*mptA* gene. The arrows marked P1 and P4 locate the primers used for the PCR analysis to confirm the absence of Cg-*mptA*. Distances are not drawn to scale. The results of the PCR analysis with the primer pair P1/P4 are shown on the right. Amplification products obtained from the wild type (wt) were applied in the left lane and that of the deletion mutant (Δ) in the right lane. ‘St’ marks the standard, where the arrowheads located at 1.5, 1 and 0.5 kb.

In order to advance further our understanding of glycosyltransferases in *Corynebacterianeae*, we focused on the genes annotated by NCgl2093 (1527 bp) and *Rv2174* (1548 bp) from *C. glutamicum* and *M. tuberculosis*, respectively, which are recognized as glycosyltransferases of unknown function (Liu and Mushegian, 2003). As shown in Fig. 2A the genomic organization of these genes in all *Corynebacterianeae* analysed is syntenic, and even in *M. leprae* the locus organization is retained, indicating an apparent fundamental function of its product. A pfam analysis (Bateman *et al*., 2004) of the ORF upstream of *Rv2174* revealed that the gene product derived bears structural similarities to polyprenyl synthetases, which could be functionally related to the glycosyltransferase, and both genes might form a transcriptional unit.

In *C. glutamicum* and *M. tuberculosis,* a number of orthologous GT-C family glycosyltransferases have been identified by us and others, which transverse the membrane (Alderwick *et al*., 2006; Kaur *et al*., 2006; Morita *et al*., 2006; Seidel *et al*., 2007a, b). Indeed, NCgl2093 and its *M. tuberculosis* orthologue (Rv2174) are putative membrane-bound GT-C glycosyltransferases (Fig. 2B). Although, both orthologues have 13 transmembrane-spanning helices (TMHs), they differ from the α-mannosyltransferase (Rv1635c) involved in the mannose capping of LAM (Dinadayala *et al*., 2006) and the arabinofuranosyltransferases AftA, AftB and the Emb proteins (Alderwick *et al*., 2005; 2006; Seidel *et al*., 2007a,b), by the absence of a periplasmatic extension at the carboxy-terminus (Fig. 2B). The degree of conservation, with respect to topology and sequence among the orthologues of NCgl2093, is high within the *Corynebacterianeae*. For instance, the similarity of the *C. glutamicum* and *M. tuberculosis* protein is 58%, and with the most distant pairs among the *Corynebacterium* species, *C. glutamicum* and *C. jeikeium*, the similarity is approximately 64%. One of the most conserved regions is between TMH 3 and 4. This long loop region is schematically shown in Fig. 2B, as is part of its sequence. This sequence is reminiscent to the glycosyltransferase family GT-C-modified DXD motif, as it contains a number of basic and acidic residues, the latter shown in mutational studies to be essential for glycosyl transfer from polyprenylated phospho-sugar donors (Berg *et al*., 2005; Seidel *et al*., 2007b). Based on the results described below, the *Rv2174* gene and its orthologues was designated *mptA* (acronym for mannopyranosyltransferase A).

### Construction and growth of *C. glutamicumΔmptA*

In an attempt to delete *mptA* in *C. glutamicum*, the non-replicative plasmid pK19mobsacBΔ*mptA* was constructed carrying sequences adjacent to Cg-*mptA*. The vector was introduced into *C. glutamicum* and in several electroporation assays kanamycin-resistant clones were obtained, indicating integration of the vector into the genome by homologous recombination (Fig. 2C). The *sacB* gene enables for positive selection of a second homologous recombination event, which can result either in the original wild-type genomic organization or in clones deleted of *mptA*. Twenty-four clones exhibiting the desired phenotype of vector-loss (Kan^S^, Suc^R^) were analysed by PCR and 18 of them were found to have Cg-*mptA* excised. These numbers indicate that the loss of Cg-*mptA* is apparently not a disadvantage for viability. As a result, one clone was subsequently termed *C. glutamicum*Δ*mptA* and confirmed by PCR to have Cg-*mptA* deleted, whereas controls with *C. glutamicum* wild type resulted in the expected larger amplification product (Fig. 2C).

Growth of wild-type *C. glutamicum* and *C. glutamicum*Δ*mptA* were compared in brain–heart infusion (BHI) medium as well as salt medium CGXII (Eggeling and Bott, 2005). Both strains exhibited comparable growth rates and final cell densities grown on CGXII of 0.31 ± 0.2 h^−1^ and 29.4 ± 2.3 (OD_600_) for the two strains respectively. Thus, *C. glutamicum*Δ*mptA* does not exhibit an apparent growth defect under the conditions assayed indicating a degree of tolerance to the deletion of Cg-*mptA*. *C. glutamicum*Δ*mptA* was transformed with pVWEx-Mt-*mptA* and pVWEx-Cg-*mptA*. As expected with these complemented strains, no alteration in growth phenotype was apparent (Fig. S1).

### Chemical analysis of extracted lipoglycans

**Fig. 3:**
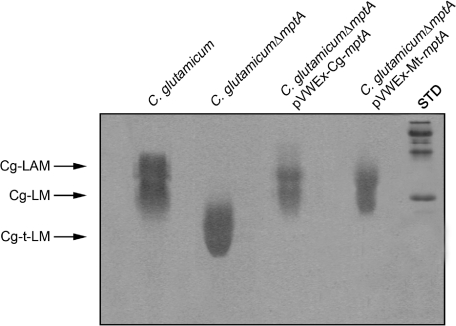
Lipoglycan profiles of *C. glutamicum*, *C. glutamicumΔmptA*, *C. glutamicum*Δ*mptA pVWEx-Cg-mptA* and *C. glutamicum*Δ*mptA pVWEx-Mt-mptA.* Lipoglycans were analysed using SDS-PAGE and visualized using a Pro-Q emerald glycoprotein stain (Invitrogen) specific for carbohydrates. The three major bands represented by Cg-LAM, Cg-LM and Cg-t-LM are indicated. The STD lane contains CandyCane glycoprotein molecular weight standards (Invitrogen). The four major bands represent glycoproteins of 180, 82, 42 and 18 kDa respectively.

Extracted lipoglycans from *C. glutamicum, C. glutamicum*Δ*mptA*, *C. glutamicum*Δ*mptA* pVWEx-Cg-*mptA* and *C. glutamicum*Δ*mptA* pVWEx-Mt-*mptA* were examined on 15% SDS-PAGE (Fig. 3). Extracts from wild-type *C. glutamicum* showed the presence of Cg-LAM and Cg-LM, while both of these lipoglycans were absent from *C. glutamicum*Δ*mptA* (Fig. 3). Interestingly, a lower-molecular-weight lipoglycan, now termed truncated (t)-LM (Fig. 3), could be observed in *C. glutamicum*Δ*mptA*. Complementation of *C. glutamicum*Δ*mptA* by either pVWEx-Cg-*mptA* or pVWEx-Mt-*mptA* restored the wild-type phenotype.

**Fig. 4:**
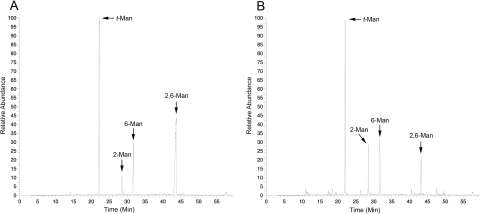
Glycosyl linkage analysis of LM from *C. glutamicum* and Cg-t-LM from *C. glutamicum*Δ*mptA*. Per-*O*-methylated samples were hydrolysed using 2 M trifluoroacetic acid, reduced and per-*O*-acetylated. The resulting partially per-*O*-methylated, per-*O*-acetylated alditol acetates from *C. glutamicum* LM (A) and *C. glutamicum*Δ*mptA* Cg-t-LM (B) were analysed by GC/MS (Tatituri *et al*., 2007).

**Fig. 6:**
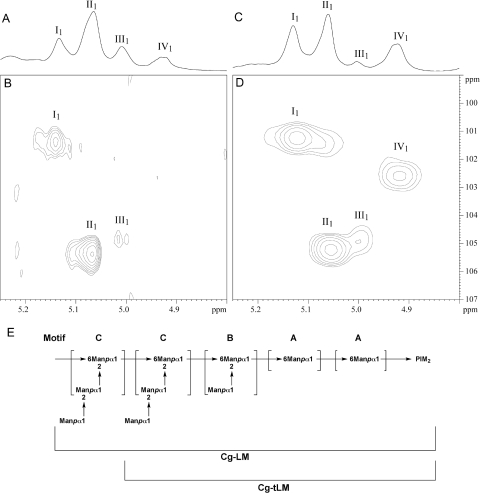
A–D. Structural characterization of LM from *C. glutamicum* (A and B) and Cg-t-LM from *C. glutamicum*Δ*mptA* (C and D). 1D ^1^H (A and C) and 2D ^1^H-^13^C HMQC (B and D) NMR spectra of Cg-LMs in D_2_O at 313K. Expanded regions (δ^1^H: 4.80–5.25) (A and C) and (δ^1^H: 4.80–5.25, δ^13^C: 99–107) (B and D) are shown. Glycosyl residues are labelled in roman numerals and their carbons and protons in Arabic numerals. I, 2,6-α-Man*p*; II, t-α-Man*p*; III, 2-α-Man*p*; IV, 6-α-Man*p*. E. Structural representation of Cg-LM and Cg-t-LM. Cg-LM contains an α(1→6)-Man*p* backbone almost completely substituted by *t*-Man*p*, *t*-Man*p*(1→2)-Man*p* units. Cg-t-LM contains a shorter α(1→6)-Man*p* backbone and a reduced degree of branching.

**Fig. 7:**
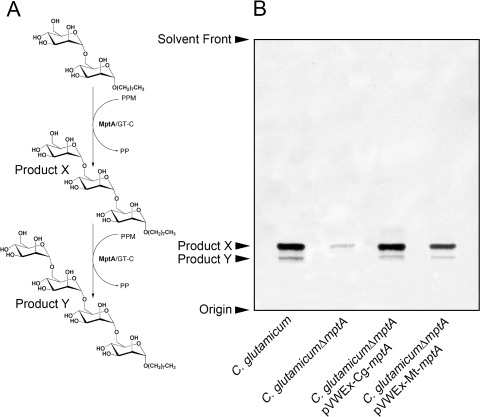
α(1→6)-Mannosyltransferase activity in membranes prepared from *C. glutamicum*, *C. glutamicum*Δ*mptA*, *C. glutamicum*Δ*mptA* pVWEx-Cg-*mptA* and *C. glutamicum*Δ*mptA* pVWEx-Mt*-mptA*. A. Biosynthetic reaction scheme of products formed in the α(1→6)-mannosyltransferase assay utilizing α-d-Man*p*-(1→6)-α-d-Man*p-O*-C_8_ and C_50_-PP[^14^C]M. B. α(1→6)-Mannosyltransferase activity determined using the synthetic α-d-Man*p*-(1→6)-α-d-Man*p-O*-C_8_ neoglycolipid acceptor in a cell-free assay using 1 mg of membrane protein as described previously (Brown *et al*., 2001). The products of the assay were re-suspended in *n*-butanol before scintillation counting. The incorporation of [^14^C]Man*p* was determined by subtracting counts present in control assays (incubations in the absence of acceptor), which were typically less than 100 cpm per assay. The remaining labelled material was subjected to TLC using silica gel plates (5735 silca gel 60F_254_, Merck) developed in CHCl_3_/CH_3_OH/H_2_O/NH_4_OH (65:25:3.6:0.5, v/v/v/v) and the products visualized by phosphorimaging (Kodak K Screen). The results represent triplicate assays in two independent experiments.

Cg-t-LM was purified by hydrophobic interaction chromatography (HIC) and compared with wild-type LM. Total sugar analysis of alditol aceteate derived sugars from Cg-t-LM by gas chromatography (GC), identified the presence of only mannose and traces of inositol (Fig. S2). Glycosyl linkage analysis of the per-*O*-methylated alditol acetate derivatives from Cg-t-LM indicated the presence of *t*-Man*p*, 2-Man*p,* 6 Man*p* and 2,6-Man*p*, similar to wild-type LM (Fig. 4A), but with an overall decrease in 6-Man*p* and 2,6-Man*p* linkages, with respect to 2-Man*p* residues (Fig. 4B). Overall, the SDS-PAGE and relative shift in glycosyl linkage analysis of Cg-t-LM illustrates that deletion of NCgl2093 results in a Cg-t-LM product that possesses a shorter mannan core and a reduced degree of branching compared with Cg-LM (Fig. 6E). This would tentatively suggest that NCgl2093 is probably involved in the synthesis of the α(1→6) mannan core via an α(1→6) mannosyltransferase, whereby deletion results in a shorter backbone and in turn branching sites. Furthermore, the analysis of Cg-t-LM in comparison with Cg-LM also suggests that the distal end to the PI of LM is probably more heavily branched (see Fig. 6E). This phenotype is in contrast to studies of Rv2181 (Kaur *et al*., 2006), whereby inactivation resulted in the complete loss of 2-Man*p* residues and a mut-LAM structure possessing a linear α(1→6) mannan core devoid of α(1→2)-Man*p* branches and as a result the characterization of Rv2181 as an α(1→2) mannosyltransferase involved in the earlier stages of LM biosynthesis (see Fig. 7).

**Fig. 5:**
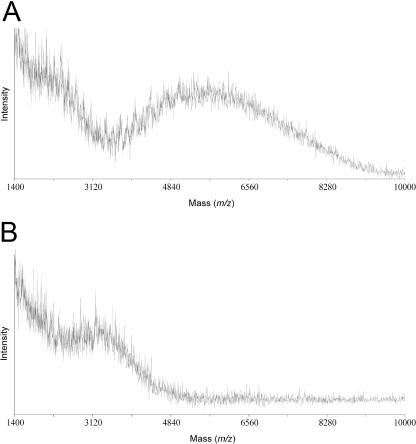
MALDI-TOF-MS spectra of LM from *C. glutamicum* (A) and Cg-t-LM from *C. glutamicum*Δ*mptA* (B). MALDI-TOF-MS spectra were acquired in the linear negative mode with delayed extraction using 2,5-dihydrobenzoic acid as a matrix.

The extracted LM from *C. glutamicum* and *C. glutamicum*Δ*mptA* were analysed by matrix-assisted laser desorption ionization time of flight mass spectrometry (MALDI-TOF-MS). The negative MALDI-TOF-MS spectrum of Cg-LM showed a broad unresolved peak centred at *m/z* 5700 (Fig. 5A), indicating a molecular mass of approximately 5.7 kDa for the major molecular species of this lipoglycan. Analysis of Cg-t-LM from *C. glutamicum*Δ*mptA* (Fig. 5B) produced a lower average molecular mass of approximately 3.3 kDa, proposing a composition based on extension of Ac_1_PIM_2_ (*m/z* 1398) (Tatituri *et al*., 2007) to afford Cg-t-LM as an average molecule centred on Ac_1_PIM_14_. As highlighted in our previous studies, the carbohydrate backbone of Cg-LM has been shown to be composed of an α(1→6)Man*p* backbone substituted at most of the O-2 positions by *t*-Man*p* and *t*-Man*p*-α-d-(1→2)-Man*p* units (Tatituri *et al*., 2007). The different nuclear magnetic resonance (NMR) spin systems of Cg-LM and Cg-t-LM were further characterized by 1D ^1^H and 2D ^1^H-^13^C Heteronuclear Multiple Quantum Correlation (HMQC) NMR (Fig. 6A–D). The Cg-t-LM from *C. glutamicum*Δ*mptA* possessed the same spin systems (Fig. 6C and D) as Cg-LM (Fig. 6A and B) and their anomeric resonances were attributed as follows: δH_1_C_1_ 5.12/101.2 (I_1_) to 2,6-Man*p*, 5.05/105.2 (II_1_) to *t*-Man*p*, 5.00/104.9 (III_1_) to 2-Man*p* and 4.92/102.6 (VII_1_) to 6-Man*p* units respectively. The intensity of 6-Man*p* unit resonances is very faint in Cg-LM 1D ^1^H-NMR spectrum (Fig. 6A) and was found to be much more intense in Cg-t-LM (Fig. 6C), allowing for the observation of a ^1^H-^13^C-NMR cross-peak at 4.92/102.6 on the HMQC NMR spectrum (Fig. 6D). Indeed, supporting our earlier glycosyl linkage analysis, integration of the 1D ^1^H-NMR resonances (2,6-Man*p* + *t*-Man*p*/6-Man*p*: 7/1 for Cg-LM and 2/1 for Cg-t-LM) indicated a reduced branching degree, approximately 50% for Cg-t-LM, as compared with 78% for Cg-LM. Altogether, the data indicate that Cg-t-LM in *C. glutamicum*Δ*mptA* occurs possibly as a result of inactivation of a core α(1→6) mannosyltransferase, presumably involved in assembly of the distal portion of Cg-LM, thereby rendering a substrate possessing reduced sites for branching (Fig. 6E).

### *In vitro* analysis of α(1 →6) mannosyltransferase activity

Initial attempts to develop an *in vitro* assay using either purified recombinant expressed Mt-MptA, Cg-MptA, or *Escherichia coli* membranes expressing the said proteins, have thus far proved unsuccessful. In an alternative approach, we assessed the capacity of membrane preparations from *C. glutamicum*, *C. glutamicum*Δ*mptA*, *C. glutamicum*Δ*mptA* pVWEx-Cg-*mptA* and *C. glutamicum*Δ*mptA* pVWEx-Mt-*mptA* to catalyse α(1→6) mannosyltransferase activity in a previously defined neoglycolipid acceptor assay utilizing an exogenous α-d-Man*p*-(1→6)-α-d-Man*p*-O-C_8_ acceptor and β-d-C_50_ polyprenyl-1-monophosphoryl-[^14^C]mannose (PP[^14^C]M) as a sugar donor (Brown *et al*., 2001) (Fig. 7A). Thin-layer chromatographic (TLC) analysis of radiolabelled products, when assayed with *C. glutamicum* membranes, resulted in the formation of two products, X and Y (Fig. 7A and B). Control assays when performed in the absence of acceptor afforded background counts, typically < 100 cpm per assay (Brown *et al*., 2001). The enzymatic synthesis of product X and Y using membranes from *C. glutamicum* relates to the biosynthesis of the radiolabelled trisaccharide α-d-[^14^C]Man*p*-(1→6)-α-d-Man*p*-(1→6)-α-d-Manp-O-C_8_ (97 864 cpm) and the tetrasaccharide α-d-[^14^C]Man*p*-(1→6)-α-d-[^14^C]Man*p*-(1→6)-α-d-Man*p*-(1→6)-α-d-Manp-O-C_8_ (5915 cpm), respectively, and is consistent with our previous studies (Brown *et al*., 2001). However, when assays were performed using membranes prepared from *C. glutamicum*Δ*mptA*, a drastically reduced amount of product X (1385 cpm) could be observed and a complete absence of product Y, indicating that Cg-MptA provides the majority of α(1→6) mannopyranosyltransferase activity utilizing the α-d-Man*p*-(1→6)-α-d-Man*p*-O-C_8_ neoglycolipid acceptor. In addition, these results also suggest the existence of a second α(1→6) mannopyranosyltransferase presumably affording the weak activity seen within the membrane preparations of *C. glutamicum*Δ*mptA* and involved in the synthesis of the α(1→6) mannan core proximal to the PI of LM. Membranes assayed with *C. glutamicum*Δ*mptA* complemented with either pVWEx-Cg-*mptA* (X, 62 953 cpm; Y, 1947 cpm) or pVWEx-Mt-*mptA* (X, 26 145 cpm; Y, 1174 cpm) restored product formation to that of wild-type *C. glutamicum*, albeit at a lower rate of transfer.

## Discussion

Apart form belonging to the supragenic taxon *Corynebacterianeae*, *M. tuberculosis* and *C. glutamicum* share common cell wall features and biosynthetic machinery. Many of the genes involved in *M. tuberculosis* cell wall and cell wall lipid biosynthesis have been shown to be essential for the growth, survival and pathogenicity of the bacillus (Belisle *et al*., 1997; Mills *et al*., 2004; Movahedzadeh *et al*., 2004; Bhatt *et al*., 2005). Due to the essentiality of such genes in mycobacteria (Sassetti *et al*., 2003), we have previously demonstrated the inherent usefulness of *C. glutamicum* in the identification of genes involved in indispensable biochemical pathways (Gande *et al*., 2004; Alderwick *et al*., 2005; 2006; Seidel *et al*., 2007a). Akin to *Corynebacterianeae* arabinogalactan biosynthesis, lipoglycan assembly is equally complex, involving many enzymes catalysing glycosyl transfer reactions producing large heterogeneous polysaccharides, in the form of LM and LAM (Nigou *et al*., 2003). The current model of LM/LAM biosynthesis involves several GT-A/B glycosyltransferases in the form of PimA, PimB and PimC, which are implicated in the initial steps of lipoglycan formation (Schaeffer *et al*., 1999; Kordulakova *et al*., 2002; Kremer *et al*., 2002). EmbC (Zhang *et al*., 2003) and Rv1635c (Dinadayala *et al*., 2006) have been shown to construct the bulk of arabinan and mannose capping of LAM respectively. The proteins participating in the intermediate steps of LM/LAM biosynthesis remain largely unresolved, apart from Rv2181, which has been proposed to be involved in α(1→2) branching in LM (Kaur *et al*., 2006). However, the linear α(1→6) backbone of LM and LAM, which serves as a core for higher polysaccharide elaboration in the form of α(1→2) branching and arabinosylation, until this report has remained unidentified.

The GT-C family of glycosyltransferases are well dispersed throughout the eukaryotes but are limited within the prokaryotes to the supragenic taxon of the Actinomycetales (Liu and Mushegian, 2003). However, due to the nature of these proteins, very little is understood regarding their protein fold, mechanism of catalysis and bioinformatic signatures (Berg *et al*., 2007). In this study, we sought to characterize the role of a putative glycosyltransferase (Rv2174) belonging to the GT-C superfamily of glycosyltransferases (Liu and Mushegian, 2003) by virtue of genomic deletion of its orthologue NCgl2093 in *C. glutamicum*. As LAM is a vital component of the *M. tuberculosis* cell wall and Rv2174 is predicted to be an essential gene (Sassetti *et al*., 2003), we utilized *C. glutamicum* as a ‘proof of principle’ model to dissect the *Corynebacteriaceae* lipoglycan biosynthetic pathway, analogous to our previous investigations regarding arabinogalactan assembly (Alderwick *et al*., 2006; Seidel *et al*., 2007a). It is not entirely clear why essential orthologous genes from *M. tuberculosis* can be deleted from *C. glutamicum*, such as *emb* (Alderwick *et al*., 2005), *aftA* (Alderwick *et al*., 2006), *aftB* (Seidel *et al*., 2007a) and *mptA* (this study). Although the structures are similar, in terms of AG and LM/LAM, a possible explanation for this paradox may be related to their respective growth rates. This seems entirely plausible when we consider *emb* mutants are readily generated in the fast-growing mycobacterial strain *M. smegmatis*, which are otherwise unattainable in slow growing *M. tuberculosis*. We present MptA as a PPM-dependent α(1→6) mannosyltransferase, involved in latter stages of LM biosynthesis, which then serves as a template for further α(1→2) branching by other α-mannosyltransferases, presumably Rv2181 (Kaur *et al*., 2006).

Our initial investigation of the extractable PIMs from *C. glutamicum*Δ*mptA* highlighted no apparent change in the profiles compared with those from *C. glutamicum* (Fig. S3), which indicated that MptA was not involved in PIM biosynthesis. This was not unsurprising, as PIM biosynthesis is completely unique to enzymes belonging to the GT-A/B glycosyltransferase family, which utilize GDP-Man*p* as a substrate (Liu and Mushegian, 2003). However, examination of lipoglycans from *C. glutamicum*Δ*mptA* afforded a complete loss of Cg-LM and Cg-LAM, and the appearance of a new smaller product (Cg-t-LM) as observed on a SDS-PAGE gel (Fig. 3). Interestingly, complementation of *C. glutamicum*Δ*mptA* with a plasmid encoding Cg-*mptA* and Mt-*mptA*, restored the lipoglycan profiles to that of wild-type *C. glutamicum* (Fig. 3). Taken together our exhaustive chemical analysis of Cg-t-LM indicates that Cg-t-LM in *C. glutamicum*Δ*mptA* occurs as a result of inactivation of a core α(1→6) mannosyltransferase, presumably involved in assembly of the distal portion of LM, thereby rendering a substrate possessing reduced sites for branching. The enzymatic activity of NCgl2093 and Rv2174 were confirmed as *bona fide*α(1→6) mannosyltransferase in a specific neoglycolipid acceptor assay (Brown *et al*., 2001).

The apparent residual glycosyltransferase activity in membranes extracted from *C. glutamicum*Δ*mptA* in the neoglycolipid assay could be attributed to another PPM-dependent GT-C mannosyltransferase, but one which has a lower efficacy for the particular acceptor used herein. This situation is entirely plausible as our evidence suggests that there are at least two α(1→6) mannosyltransferases, which utilize PPM as a substrate for glycosyl transfer, inferring that both belong to the GT-C family of glycosyltransferases (Liu and Mushegian, 2003) and are therefore involved in LM-backbone synthesis. Indeed, inspection of the NMR and glycosyl linkage data hints that MptA is involved in the latter stages of α(1→6) backbone synthesis, which is likely to be more highly elaborated with α(1→2) mannose residues compared with the PI-end of the polysaccharide which contains less branching.

**Fig. 8:**
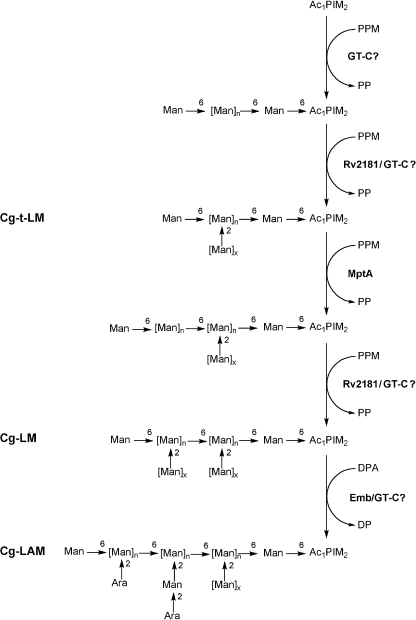
Proposed biosynthetic of lipoglycans in *Corynebacterianeae*. Ac_1_PIM_2_ represents the initiation point for further lipoglycan assembly leading to Cg-t-LM, LM and LAM. n denotes residues of unknown numbers, whereas x represents residues ranging from 0 to 2.

This system of polysaccharide biosynthesis is mirrored in the assembly of *Corynebacteriaceae* arabinogalactan, for which several glycosyltransferases are involved in priming, extension and termination of arabinan biosynthesis, in the form of AftA, AftB and the Emb proteins (Escuyer *et al*., 2001; Alderwick *et al*., 2005; 2006; Seidel *et al*., 2007a). It may be argued that in order for the complete biosynthesis of Cg-LAM, a fully functional molecule of Cg-LM must be assembled to serve as a precursor for Cg-LAM-specific arabinosyltransferases. Furthermore, the biosynthesis of LM and LAM is likely to occur in discrete steps, with each glycosyltransferase recognizing a previously synthesized acceptor molecule which is suitably ‘primed’ for the next step of molecular assembly (Fig. 8). Herein, we are proposing a biosynthetic pathway that involves MptA as an α(1→6) mannosyltransferase responsible for the latter stages of LM-backbone biosynthesis, and sheds further light on the complexities of *Corynebacteriaceae* cell wall biosynthesis as a potential drug target.

## Experimental procedures

### Bacterial strains and growth conditions

*Corynebacterium glutamicum* ATCC 13032 (the wild-type strain, and referred to for the remainder of the text as *C. glutamicum*) and *Escherichia coli* DH5αmcr were grownin Luria–Bertani broth (LB, Difco) at 30°C and 37°C respectively. The recombinant strains generated in this study were grown on complex BHI medium (Difco), and the salt medium CGXII used for *C. glutamicum* as described (Eggeling and Bott, 2005). Kanamycin and ampicillin were used at a concentration of 50 μg ml^−1^. Samples for lipid analyses were prepared by harvesting cells at an optical density (OD) of 10–15, followed by a saline wash and freeze drying. *M. tuberculosis* H37Rv DNA was obtained from Dr J.T. Belisle at the NIH Tuberculosis Research Materials and Vaccine Testing Contract at Colorado State University. All other chemicals were of reagent grade and obtained from Sigma-Aldrich.

### Construction of plasmids and strains

In order to enable deletion of the gene with the locus tag *C. glutamicum* NCgl2093 (Cg-*mptA*) the primer pair P1, CGCTTCTAGACAACGCGCTGATAAGCAATCTCC (all primers given in 5′ to 3′ direction) and P2rev, CCCATCCACTAAACTTAAACACGTTGAAAAAGTGT**CAT**ACGCG, were used with start codon in bold and restriction endonuclease sites underlined to generate a 288 bp fragment upstream of NCgl2093. Similarly the pair P3, TGTTTAAGTTTAGTGGATGGGACTGACCCTGCAACAAC, and P4rev, GCGGGAATTCGAAGGAAAACACCAACCGTTTCATC, was used to generate a 340 bp downstream fragment. Using both isolated fragments cross-over PCR was applied with primers P1 and P4rev to generate a 628 bp fragment which was cloned into EcoRI–XbaI-cleaved pK19mobsacB (Schafer *et al*., 1994) resulting in pK19mobsacBΔ*mptA*.

To enable plasmid encoded expression of *C. glutamicum* NCgl2093 (Cg-*mptA*), the gene was amplified using the primer pairs 2093for, CGCGTCAT**ATG**ACACTTTTTCAACGTTTAACCAAC, and 2093rev, GTAATGGA**TCC**TAGGAAACGGTATGCGGGGAG, with start and stop codons in bold and restriction endonuclease sites underlined. The resulting fragment was cloned into pGEM-T, excised as an NdeI–BamHI fragment and inserted into NdeI–BamHI-cleaved pET16b to result in pET16b-NCgl 2093 for expression studies in *E. coli*. For expression in *C. glutamicum* the primer pair 2093rev together with 2093RBSfor, GCGCGGTTAACAGGGAGATATAG**ATG**ACACTTTTTCAACGTTTAAC, was used. The resulting fragment was cloned into pGEM-T, excised as an HpaI–SpeI fragment and cloned into the *E. coli–C. glutamicum* shuttle vector pVWEx resulting in pVWEx-Cg-*mptA*.

To clone Rv2174 of *M. tuberculosis* (Mt-*mptA*) the primer pairs Rv2174for, CATCTACAT**ATG**ACTACTCCGAGCCATGCTCCAGC, and Rv2174rev, CAGTGAGAT**CTC**TATGGCGTATTGACCACCG, were used. The amplificate was cloned into pGEM-T as above and inserted into pET16b resulting in pET16b-Rv2174 for expression studies in *E. coli*. For expression in *C. glutamicum* the primer pair 2174rev together with 2174RBSfor, CACTAGTTAACAGGGAGATATAG**ATG**ACTACTCCGAGCCATG, was used. The resulting fragment was cloned as above into pGEM-T and subcloned resulting in pVWEx-Mt-*mptA*. All plasmids used were confirmed by sequencing for integrity.

For the chromosomal deletion of Cg-*mptA*, plasmid pK19mobsacBΔ*mptA* was used, taking advantage of the kanamycin resistance gene *aph*, to select for plasmid integration in the first round of homologuous recombination and the sucrose gene *sacB*, to select for loss of vector in the second round of homologuous recombination (Jager *et al*., 1992). The successful deletion in the resulting strain *C. glutamicum*Δ*mptA* was verified by use of two different primer pairs. The amplification with primers CGGCCGCTTACACGATTGCGC (P1) and CGCAGAAATACCCTAAAGATTCTCCATTAGAGC (P4), is shown in Fig. 2C, giving the expected sizes of 2505 bp in the wild type and 1050 bp in the deletion mutant. Plasmid pVWEx-Cg-*mptA* and pVWEx-Mt-*mptA* were introduced into *C. glutamicum*Δ*mptA* by electroporation with selection to kanamycin resistance (25 μg ml^−1^).

### Lipid extraction and analysis

Polar lipids and apolar lipids were extracted as described previously (Tatituri *et al*., 2007). Briefly, 6 g of dry *C. glutamicum* cells were treated in 220 ml of methanolic saline (20 ml of 0.3% NaCl and 200 ml of CH_3_OH) and 220 ml of petroleum ether for 2 h. The suspension was centrifuged and the upper layer containing apolar lipids was separated. An additional 220 ml of petroleum ether was added, mixed and centrifuged as described above. The two upper petroleum ether fractions were combined and dried. For polar lipids, 260 ml of CHCl_3_/CH_3_OH/0.3% NaCl (9:10:3, v/v/v) was added to the lower aqueous phase and stirred for 4 h. The mixture was filtered and the filter cake re-extracted twice with 85 ml of CHCl_3_/CH_3_OH/0.3% NaCl (5:10:4, v/v/v). Equal amounts of CHCl_3_ and 0.3% NaCl (145 ml each) were added to the combined filtrates and stirred for 1 h. The mixture was allowed to settle, and the lower layer containing the polar lipids recovered and dried. The polar lipid extract was examined by two-dimensional TLC on aluminum-backed plates of silica gel 60F_254_ (Merck 5554), using CHCl_3_/CH_3_OH/H_2_O (65:25:4, v/v/v) in the first direction and CHCl_3_/CH_3_COOH/CH_3_OH/H_2_O (40:25:3:6, v/v/v/v) in the second direction. Glycolipids were visualized by either spraying plates with α-naphthol/sulphuric acid or 5% ethanolic molybdophosphoric acid followed by gentle charring of plates. Identification of phospholipids was carried out using the Dittmer and Lester reagent as described in Tatituri *et al*. (2007).

### Extraction and purification of lipoglycans

Lipoglycans were extracted from previously delipidated cells (lipid extraction and analysis) as previously described (Nigou *et al*., 1997; Ludwiczak *et al*., 2002). Briefly, cells were broken by sonication (MSE Soniprep 150, 12 micron amplitude, 60 s ON, 90 s OFF for 10 cycles, on ice) and the cell debris refluxed five times with 50% C_2_H_5_OH at 68°C, for 12 h intervals. The cell debris was removed by centrifugation and the supernatant containing lipoglycans, neutral glycans and proteins dried. This dried extract was then treated with hot phenol-H_2_O. The aqueous phase was dialysed against H_2_O (5 l) using a 1500 MWCO membrane (Spectrapore) and dried, followed by extensive treatments with α-amylase, DNase, RNase chymotrypsin and trypsin. The fraction was dialysed once again in H_2_O (5 l) to remove residual impurities and enzymes.

The crude lipoglycan extract was dried and re-suspended in buffer A (50 mM ammonium acetate and 15% propan-1-ol) and subjected to Octyl Sepharose CL-4B HIC (2.5 cm × 50 cm) (Leopold and Fischer, 1993). The column was washed initially with 4 column volumes of buffer A to ensure removal of neutral glycans followed by buffer B (50 mM ammonium acetate and 50% propan-1-ol). The eluent was collected and concentrated to approximately 1 ml and precipitated using 5 ml of C_2_H_5_OH. The sample was dried using a Savant Speedvac and then re-suspended in buffer C [0.2 M NaCl, 0.25% sodium deoxycholate (w/v), 1 mM EDTA and 10 mM Tris-HCl, pH 8] to a final concentration of 200 mg ml^−1^. The sample was gently mixed and left to incubate for 48 h at room temperature. The sample was then loaded onto a 200 ml Sephacryl S-200 column previously equilibrated with buffer C. The sample was eluted with 400 ml of buffer C at a flow rate of 3 ml h^−1^, collecting 1.5 ml fractions. The fractions were monitored by SDS-PAGE using either a silver stain utilizing periodic acid and silver nitrate (Hunter *et al*., 1986) or a Pro-Q emerald glycoprotein stain (Invitrogen) and individual fractions pooled and dialysed extensively against buffer D (10 mM Tris-HCl, pH 8, 0.2 M NaCl, 1 mM EDTA) using a 1500 MWCO membrane (Spectrapore) for 72 h with frequent changes of buffer. The samples were further dialysed against deionized water for 48 h using a 1500 MWCO membrane (Spectrapore) with frequent changes of water, lyophilized and stored at −20°C.

### Glycosyl compositional and linkage analysis

Lipoglycans were hydrolysed using 2 M trifluoroacetic acid, reduced with NaB^2^H_4_, and the resultant alditols per-*O*-acetylated before examination by GC (Tatituri *et al*., 2007). Glycosyl linkage analyses were performed as described previously (Tatituri *et al*., 2007). Briefly, lipoglycan samples were per-*O*-methylated using dimethyl sulphinyl carbanion, hydrolysed using 2 M trifluoroacetic acid, reduced using NaB^2^H_4_ and per-*O*-acetylated. The resulting per-*O*-methylated alditol acetates were solubilized in CHCl_3_ before analysis by gas chromatography/mass spectrometry (GC/MS) (Tatituri *et al*., 2007). GC analysis was performed using a Thermoquest Trace GC 2000. Samples were injected in the splitless mode. The column used was a DB225 (Supelco). The oven was programmed to hold at an isothermal temperature of 275°C for a run time of 15 min. GC/MS was carried out on a Finnigan Polaris/GCQ PlusTM. The column used was a BPX5 (Supleco). Injector temperature was set at 50°C, held for 1 min and then increased to 110°C at 20°C min^−1^. The oven was held at 110°C then ramped to 290°C at 8°C min^−1^ and held for 5 min to ensure all the products had eluted from the column. All the data were collected and analysed using Xcaliber (v.1.2) software.

### MALDI-TOF-MS analyses

The matrix used was 2,5-dihydroxybenzoic acid at a concentration of 10 μg μl^−1^, in a mixture of water/ethanol (1:1, v/v), 0.1% trifluoroacetic acid. LM samples (0.5 μl) at a concentration of 10 μg μl^−1^ were mixed with 0.5 μl of the matrix solution. Analyses were performed on a Voyager DE-STR MALDI-TOF instrument (PerSeptive Biosystems, Framingham, MA) using linear mode detection. Mass spectra were recorded in the negative mode using a 300 ns time delay with a grid voltage of 80% of full accelerating voltage (25 kV) and a guide wire voltage of 0.15%. The mass spectra were mass assigned using external calibration.

### NMR spectroscopy

Nuclear magnetic resonance spectra of LM samples were recorded on a Bruker DMX-500 equipped with a double resonance (1H/X)-BBi z-gradient probe head. All samples were exchanged in D_2_O (D, 99.97% from Euriso-top, Saint-Aubin, France), with intermediate lyophilization, and then dissolved in 0.5 ml of D_2_O and analysed at 313K. The ^1^H and ^13^C NMR chemical shifts were referenced relative to internal acetone at 2.225 and 34.00 p.p.m. respectively. All the details concerning NMR sequences used and experimental procedures were described previously (Gilleron *et al*., 1999; 2000).

### *In vitro* analysis of α(1 →6) mannosyltransferase activity

*Corynebacterium glutamicum*, *C. glutamicum*Δ*mptA*, *C. glutamicum*Δ*mptA* pVWEx-Cg-*mptA* and *C. glutamicum*Δ*mptA* pVWEx-Mt-*mptA* were cultured to the mid-logarithmic growth phase in 1 l of BHIS medium supplemented with kanamycin and 0.2 mM IPTG where appropriate. Cells were harvested by centrifugation, re-suspended in 20 ml of buffer E (50 mM MOPS pH 7.9, 5 mM 2-mercaptoethanol and 5 mM MgCl_2_) and lysed immediately by sonication (60 s ON, 90 s OFF for a total of 10 cycles). The lysate was clarified by centrifugation at 27 000 *g* (4°C, 30 min) and membranes were deposited by centrifugation of the supernatant at 105 000 *g* (4°C, 90 min). The membranes were re-suspended in buffer E to a final protein concentration of 10–15 mg ml^−1^. The neoglycolipid acceptor α-d-Man*p*-(1→6)-α-d-Man*p*-O-C_8_ (stored in C_2_H_5_OH) and C_50_-PP[^14^C]M [stored in CHCl_3_/CH_3_OH, 2:1, v/v, and prepared as described in Gurcha *et al*. (2002)] were separated into aliquots into 1.5 ml eppendorf tubes to a final concentration of 2 mM and 0.25 μCi (0.305 Ci mmol^−1^), respectively, and dried under nitrogen. IgePal CA-630 (Sigma Aldrich) was added (0.1%, v/v) with appropriate amounts of buffer E (final volume 80 μl). Tubes were sonicated for 15 min to re-suspend the lipid-linked components and then mixed thoroughly with the remaining assay components, which included 1 mM ATP, 1 mM NADP, and membrane protein (1 mg) from either *C. glutamicum*, *C. glutamicum*Δ*mptA*, *C. glutamicum*Δ*mptA* pVWEx-Cg-*mptA* or *C. glutamicum*Δ*mptA* pVWEx-Mt-*mptA*. Assays were initiated and incubated at 37°C for 1 h. The reaction was quenched with the addition of 533 μl of CHCl_3_/CH_3_OH (1:1, v/v). After mixing and centrifugation at 27 000 *g* for 15 min at 4°C, the supernatant was removed and dried under nitrogen. The residue was then re-suspended in 700 μl of C_2_H_5_OH/H_2_O (1:1, v/v) and loaded onto a 1 ml SepPak strong anion exchange cartridge (Supleco) pre-equilibrated with C_2_H_5_OH/H_2_O (1:1, v/v). The column was washed with 2 ml of C_2_H_5_OH, and the eluate collected, dried and partitioned between the two phases arising from a mixture of *n*-butanol (3 ml) and water (3 ml). The resulting organic phase was recovered after centrifugation at 3500 *g*, and the aqueous phase was again extracted twice with 3 ml of water-saturated butanol. The pooled extracts were back-washed twice with *n*-butanol saturated water (3 ml). The *n*-butanol fraction was dried and re-suspended in 200 μl of *n*-butanol. The extracted radiolabelled material was quantified by liquid scintillation counting using 10% of the labelled material and 5 ml of EcoScintA (National Diagnostics, Atlanta, GA). The incorporation of [^14^C]Man*p* was determined by subtracting counts present in control assays (incubations in the absence of acceptor), which were typically less than 100 cpm per assay. The remaining labelled material was subjected to TLC using silica gel plates (5735 silca gel 60F_254_, Merck) developed in CHCl_3_/CH_3_OH/H_2_O/NH_4_OH (65:25:3.6:0.5, v/v/v/v) and the products visualized by phosphorimaging (Kodak K Screen).

## Supplementary material

The following supplementary material is available for this article:

Fig. S1 Growth phenotype of C. glutamicum, C. glutamicum-ΔmptA and complements.

Fig. S2 Glycosyl compositional analysis of Cg-LM (C. glutamiocum) and Cg-t-LM (C. glutamicumΔmptA).

Fig. S3 Analysis of PIM biosynthesis in C. glutamicum and C. glutamicumΔmptA.

This material is available as part of the online article from: http://www.blackwell-synergy.com/doi/abs/10.1111/j.1365-2958.2007.05884.x (This link will take you to the article abstract).
