# Supplementary material for: Identification of an α(1→6) mannopyranosyltransferase (MptA), involved in Corynebacterium glutamicum lipomanann biosynthesis, and identification of its orthologue in Mycobacterium tuberculosis
Source: Mol Microbiol. 2007 Sep;65(6):1503–17. doi: 10.1111/j.1365-2958.2007.05884.x (PMC2157549; doi:10.1111/j.1365-2958.2007.05884.x)
Supplement: Fig. S1 — Growth phenotype of C. glutamicum, C. glutamicum-ΔmptA and complements. [file mmi0065-1503-SD1.pdf]

Supplementary Figure 1

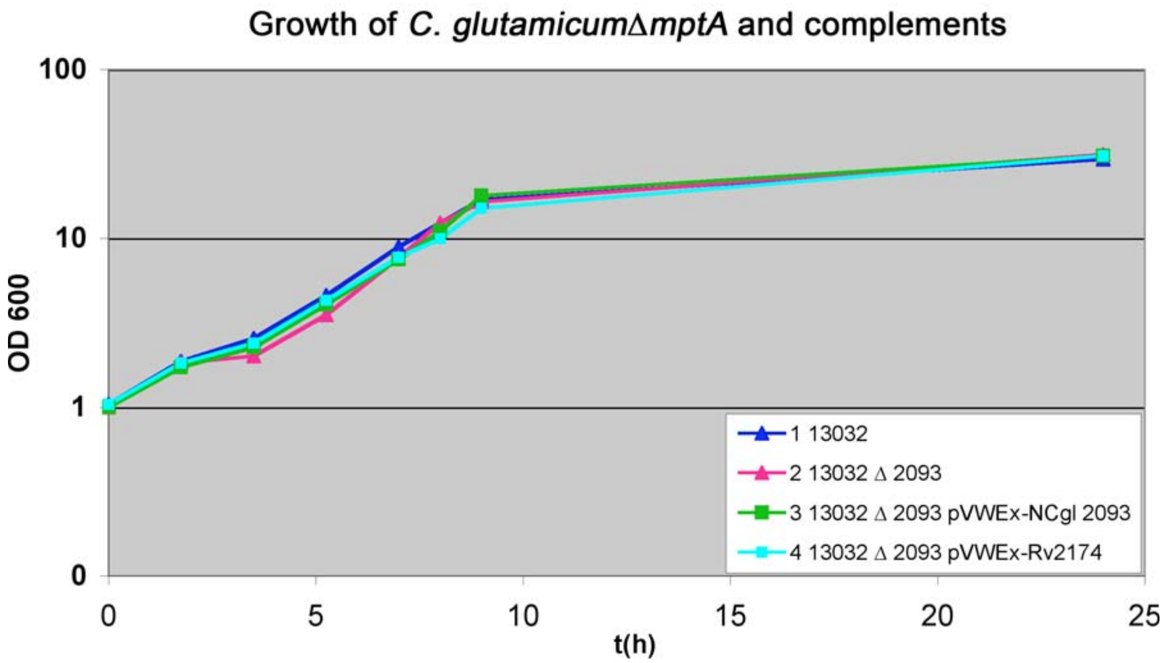

Supplementary Fig.1: ***Growth phenotype of C. glutamicum, C. glutamicumΔmptA and complements.*** Consequences of *mptA* deletion on growth in rich medium (BHI).

Supplementary Figure 2

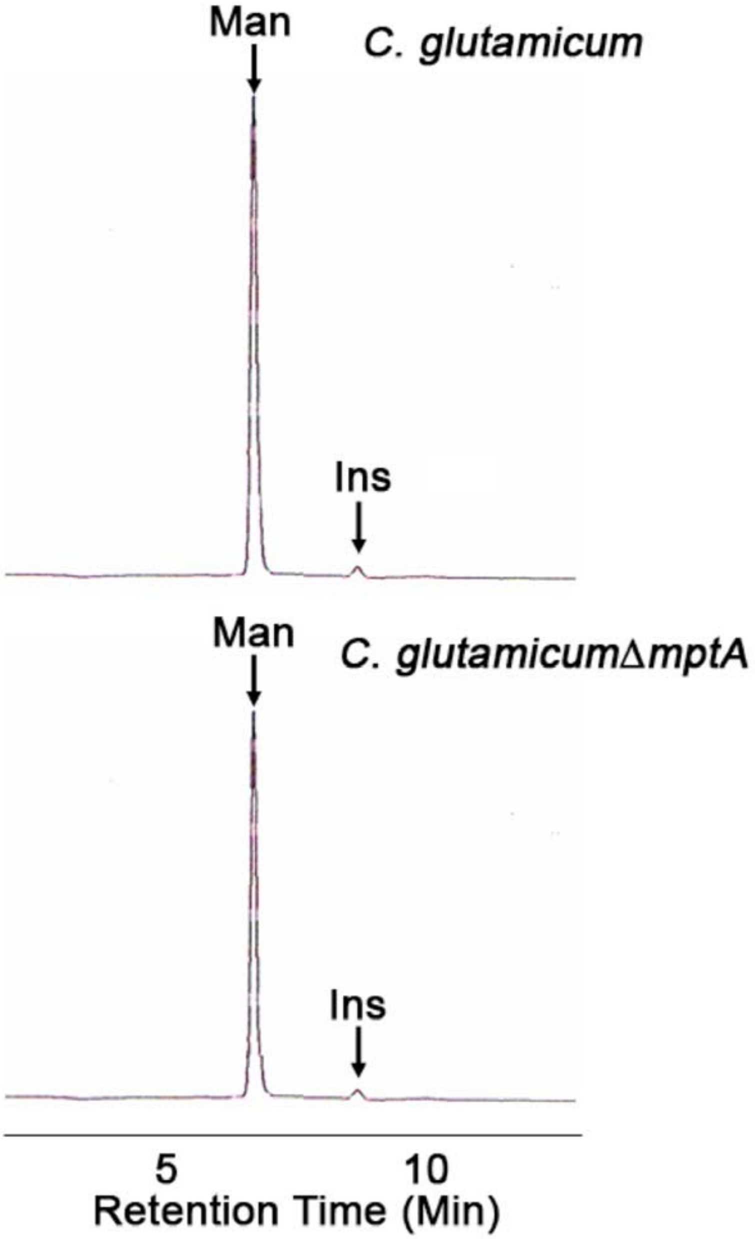

Supplementary Fig 2: ***Glycosyl compositional analysis of Cg-LM (C. glutamiocum) and Cg-t-LM (C. glutamicumΔmptA).*** Samples of purified lipoglycans were hydrolysed with 2 M trifluoroacetic acid, reduced, per-*O*-acetylated, and subjected to gas chromatography as described in Tatituri *et al*. (2007) and compared to known standards.

Supplementary Figure 3

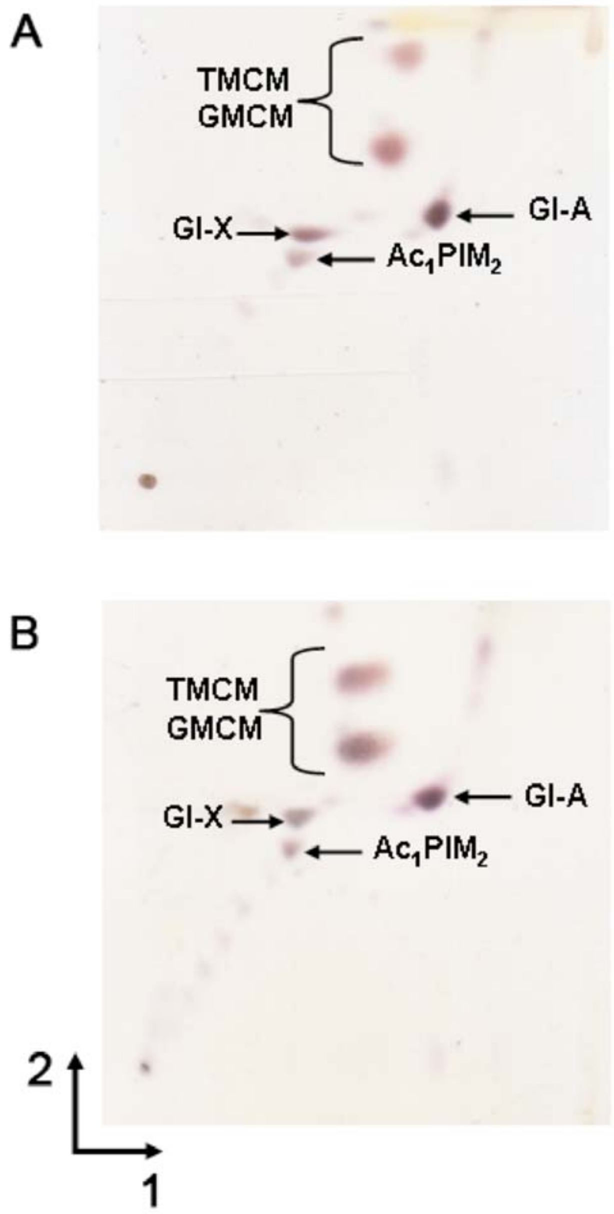

Supplementary Fig 3: ***Analysis of PIM biosynthesis in C. glutamicum and C. glutamicumΔmptA.*** The polar lipid profiles of *C. glutamicum* **(A)** and *C. glutamicum*Δ*mptA* **(B)** are shown. The polar lipid extract was examined by two-dimensional thin layer chromatography on aluminum-backed plates of silica gel 60 F_{254} (Merck 5554), using $CHCl_3/CH_3OH/H_2O$ (65:25:4, v/v/v) in the first direction and $CHCl_3/CH_3COOH/CH_3OH/H_2O$ (40:25:3:6, v/v/v/v) in the second direction Tatituri *et al*. (2007). Glycolipids were visualized by spraying plates with α-naphthol/sulfuric acid, followed by gentle charring of the plates.
